# Silk-Sericin Release from Polymeric Scaffold as Complementary Dermocosmetic Treatment for Acne

**DOI:** 10.3390/polym17060781

**Published:** 2025-03-14

**Authors:** Arianna Vargas González, Patricia Pérez Ramos, Eva María Pérez-Soriano, Francisco Javier Sola Dueñas, Denise Pérez Almazán, Jomarien García Couce, Gastón Fuentes Estévez

**Affiliations:** 1 Instituto de Farmacia y Alimentos (IFAL), Universidad de La Habana, Havana 13100, Cuba; vargasarianna250@gmail.com (A.V.G.); patry@ifal.uh.cu (P.P.R.); 2 Centro de Biomateriales, Universidad de La Habana, Havana 10400, Cuba; francisco.sola@biomat.uh.cu (F.J.S.D.); jgcouce@biomat.uh.cu (J.G.C.); gastonfe@biomat.uh.cu (G.F.E.); 3 Departamento de Ingeniería y Ciencia de los Materiales y del Transporte, Escuela Politécnica Superior, Universidad de Sevilla, 41011 Seville, Spain; 4 Centro Investigaciones de Plantas Proteicas y Productos Bionaturales (CIPB), Complejo Barlovento, Havana 11300, Cuba; denisepereza@yahoo.es

**Keywords:** acne, dermocosmetics, polymer scaffold, silk sericin

## Abstract

Currently, acne therapy relies not only on specific drugs but also on complementary treatments, such as dermocosmetics. Several studies have reported the use of chitosan and alginate in scaffolds for drug delivery systems. These materials can be loaded with a product that exhibits anti-acne properties such as silk sericin, a protein with antioxidant, photoprotective, and moisturizing properties. Therefore, this study proposes the development of a chitosan/alginate scaffold, loaded with sericin, to serve as a dermocosmetic platform complementing the pharmacological treatment of acne. The moisture content of the alginate and chitosan was determined as 14.7 and 21%, respectively; the ash content, which is similar for both polymers, was approximately 5%. The employed chitosan had a deacetylation degree of 82%, as determined by infrared spectrometry and corroborated by potentiometry. This technique was also used to determine the mannuronic/guluronic ratio of the alginate [M/G = 1.3] and confirm the identity of each one of the polymers in the raw materials and the resulting scaffolds. The molecular weights of alginate, chitosan, and sericin were 85, 5.1, and 57.4 kDa, respectively. The pH [6.31] and total protein concentration of the sericin solution [c(SER) = 6.1 mg/mL] were determined using UV-visible spectrophotometry. Swelling and release studies indicated that, although there were varying degrees of cross-linking and certain variables to control, the mechanism that defines the nature of both processes (otherwise complementary) is the relaxation of the polymer chains.

## 1. Introduction

Biopolymers are large biocompatible, sometimes biodegradable, macromolecules that can be obtained from natural or synthetic sources. For this reason, their medical and pharmaceutical applications currently constitute one of the greatest fields of interest in macromolecules development, due to their use as cardiovascular, orthopedic, ophthalmological and dental therapeutic devices, skin substitutes, drug delivery systems, and sensors for diagnostic purposes [1]. Their biocompatibility favours the selection of pharmaceutical formulations since they are less likely to produce an allergic reaction than other compounds [2].

Several studies have reported on the use of chitosan and alginate in the fabrication of porous three-dimensional structures (scaffolds) for tissue engineering, achieving materials with enhanced mechanical properties when cross-linking techniques are applied [3]. Due to their biocompatibility, biodegradability, and ability to form three-dimensional structures through various techniques for the controlled release of active ingredients, the biopolymers alginate and chitosan are an ideal combination for developing an application device for dermocosmetic purposes.

Sericin, one of the two substances that make up the structure of the Bombyx mori silkworm cocoon, is a biocompatible and biodegradable natural protein with potential in food and cosmetic industries, due to its antioxidant, antityrosinase, antibacterial, and antifungal activity [4].

On the other hand, acne is a chronic inflammatory disease of the pilosebaceous unit resulting from androgen-induced increased sebum production, impaired keratinization, inflammation, and bacterial colonization of the hair follicles of the face, neck, chest, and back by *Cutibacterium acnes* [5]. Its treatment must be individualized, considering age, sex, clinical form, intensity, social circumstances of the patient, and disease impact on their quality of life [6]. This can be classified as pharmacological or non-pharmacological depending on its association with the use of medications [7].

Modern acne therapy is based not only on the use of specific drugs, but also on complementary treatments such as dermocosmetics, mainly moisturizing products, cleansers, and sunscreens [8]. The use of treatments derived from natural plants or phytotherapeutics as an alternative or adjuvant to conventional treatments is attractive to patients due to their safety and minimal risk of side effects [9]. For these reasons, this study aimed to develop a porous sponge from natural polymers (alginate and chitosan) designed to serve as a delivery system for a sericin solution. The sericin-loaded sponge could serve as an adjuvant for traditional acne treatments, due to its biocompatibility and the beneficial properties of the sericin, which makes it an ideal active ingredient. Additionally, the device is designed to be reusable through simple washing in an alcoholic solution, reducing waste and enhancing environmental sustainability. This aligns with the core objectives of our investigation, developing a sustainable topical treatment while minimizing environmental impact.

## 2. Materials and Methods

### 2.1. Materials

All chemicals were of analytical grade and used as received. Chitosan was extracted from shrimp exoskeleton and provided by the National Autonomous University of Mexico (UNAM; Mexico City, Mexico). Sodium alginate was obtained from BDH Chemicals Ltd. Poole (London, UK). Polysorbate 80 (Tween 80), hydrochloric acid (HCl), and acetic acid (CH_3_-COOH) were obtained from Merck GmbH (Darmstadt, Germany). Sodium hydroxide (NaOH) was purchased from Uni-Chem (Mumbai, India). Bovine serum albumin (BSA) was obtained from Boehringer Manheim GmbH (Manheim, Germany). EDC [1-ethyl-3-(3-dimethylaminopropyl) carbodiimide] and NHS (*N*-hydroxysuccinimide) were used for the scaffold crosslinking and provided by Nacalai Tesque Inc. (Kyoto, Japan) and Wako Pure Chemical Industries (Osaka, Japan), respectively. Silkworm cocoons (*Bombyx mori*) were cultivated and supplied by the Research Centre for Protein Plants and Bionatural Products (CIPB; Havana, Cuba).

### 2.2. Extraction of Sericin

The selected cocoons were cut into small irregular pieces (<10 mm^2^) and the degumming technique was used, at 100 °C with distilled water, in a ratio of 1:50 (g of cocoons/mL water). The sericin solution obtained was filtered to eliminate particulate material and possible impurities present in the solution, and then stored at 4 °C.

### 2.3. Materials Characterization

#### 2.3.1. Fourier Transform Infrared Spectroscopy with Attenuated Total Reflection (ATR-FTIR)

An infrared spectrophotometer Bruker Tensor 27 from Bruker Corp (Billerica, MA, USA) was used and the ATR-FTIR analysis was performed ranging from 4000 to 400 cm^−1^ with 32 scans and 4 cm^−1^ of resolution using the KBr method (1:100 ratio). To prepare the KBr disk, 2 mg of the dry sample was placed in an agate mortar, crushed perfectly until it adhered to the walls of the mortar, then the halide (KBr) was added and ground again until perfectly homogenized. This mixture was compressed under sufficient pressure to obtain a transparent tablet.

#### 2.3.2. Humidity Percentage

Approximately 1 g of polymer was weighed on the humidity balance Moisture Analyzer Digital Xy-100MM (Grand Island, NY, USA). The moisture percentage value (%H) was registered.

#### 2.3.3. Ash Content

Approximately 1 g of polymer was weighed and placed in a muffle furnace model Shanghai Pudong Rongfeng Scientific Instrument Co. (Shanghai, China) at a temperature of 900 °C for 6 h. After this time, it was covered and allowed to cool to room temperature in a desiccator. Each crucible was weighed, and the procedure was repeated every 30 min until the measurement remained constant.

#### 2.3.4. *M/G* Ratio of Sodium Alginate

The estimation of the absorbance ratio at 1290 cm^−1^ and 1320 cm^−1^ allowed the determination of the *M/G* fraction with an error of 3% using Equation (1) [10].

(1) $$\frac{M}{G}=\frac{A_{1320}}{A_{1290}}$$

where *M/G* is the mannuronic acid (*M*)/guluronic acid (*G*) ratio of the alginate and *A* is the absorbance (at 1320 cm^−1^ and 1290 cm^−1^, respectively).

#### 2.3.5. Deacetylation Degree (DD) of Chitosan

The DD was determined by potentiometry with a Crison Basic 20 pH metre (Crison Instruments, Barcelona, Spain). Chitosan was dissolved in a known excess of hydrochloric acid (HCl) which was titrated with a standardized solution of sodium hydroxide (NaOH) 0.25 M. For the standardization of potassium biphthalate, an exact concentration (0.5 N) was used and prepared using carbon dioxide-free water as the solvent. In the titration, a curve of pH versus NaOH volume added was obtained. The DD was calculated according to the following equation:

(2) $${\%NH}_{2}=\frac{16.1\cdot (y-x)}{w\cdot f}$$

where *%NH*_2_ is the degree of deacetylation; *y* is the major inflection point and *x* is the minor; *w* is the dry mass of chitosan and *f* is the molarity of NaOH. The DD was also confirmed by ATR-FTIR. From the absorbance values obtained by FTIR, the degree of acetylation (*%DA*) of chitosan was determined. This was followed and corroborated by ^1^H NMR [11]. For this, Equation (3) was used.

(3) $$\%DA=\frac{\frac{A_{1320}}{A_{1420}}-0.3822}{0.03133}$$

This formula is based on the absorption bands at 1320 cm^−1^ and 1420 cm^−1^. The first band is characteristic of the amine function, while the second corresponds to the acetylated amide and is chosen as the reference band.

#### 2.3.6. Hydrodynamic Radius and Molecular Weight

A Dynamic Light Scattering instrument Litesizer^TM^ 500 (Anton Paar GmbH, Graz, Austria) was used. Tests were performed at 25 °C, at a measurement lateral angle of 90°. The Kalliope Professional programme (Anton Paar GmbH, Graz, Austria; version 2.18) was used to acquire the results.

To determine the hydrodynamic radius of the polymers, the Dynamic Light Scattering (DLS) technique was carried out repeatedly with an equilibrium time of 1 min, using a low-volume quartz cuvette with 50 μL of sample at a concentration of 1 mg/mL. Both sample and solvent were filtered through a cellulose acetate membrane with a pore size of 0.45 μm prior to use. The repetition series consisted of 5 consecutive measurements.

For the molecular weight determination by Static Light Scattering (SLS), the same conditions were established. A spherical correction was chosen and, for the hydrodynamic radius, the one obtained with the DLS analysis was selected. Toluene was used as a reference.

#### 2.3.7. Sericin Solution Characterization (Total Protein Concentration and pH)

A calibration curve in the range 0.2–1.0 mg/mL was designed with bovine serum albumin (BSA). The absorbance was measured (*λ* = 280 nm, slope = 0.613, *R*^2^ = 99.99%) by a multimode microplate reader for absorbance Tecan Infinite 200 Pro (Tecan Austria GmbH, Grödig, Austria). The pH determination was developed with a Crison Basic 20 pH metre (Barcelona, Spain) with a scale from −2 to 16 and error ± 0.02 pH units, at 25 °C.

### 2.4. Polymer Solutions Preparation

Aqueous solutions of alginate (2% *w*/*v*) and chitosan (1.25% *w*/*v*) were prepared in distilled water. To this end, 5 g of polymer were dissolved in 250 mL and 400 mL of distilled water, respectively, and stirred for 24 h by a magnetic stirrer Janke and Kunkel and Ikamag RH (Staufen, Germany). Then, acetic acid (1% *w*/*v*) was added to the chitosan dissolution, which was left stirring for an additional 24 h.

### 2.5. Scaffold Preparation

To obtain the polymer emulsion, equivalent volumes of 1.25% aq. chitosan dissolution and 2% aq. alginate dissolution were mixed under stirring (5000 rpm, Ultra-Turrax stirrer (IKA, Staufen, Germany)). Then, 1 mL of Tween 80 per 40 mL of polymer mixture was added and the stirring was maintained for 2 min. The mixture was poured into moulds (50 × 12 mm) and then frozen at −80 °C for 24 h. Finally, the scaffold was freeze-dried in a Labconco freeze-dryer (Labconco Corp., Kansas City, MO, USA) [12].

EDC and NHS dissolutions were prepared and used as cross-linking reagents. The EDC–NHS molar ratio (5:1) was maintained and the overall concentration of the cross-linking agent was varied (0.0%, 0.5%, 1.0%, and 1.5%) and dissolved in 95% ethanol. The samples were covered with the solutions for 4 h. Afterward, they were washed three times with 95% ethanol, frozen for 24 h, and lyophilized over the same period.

### 2.6. Scaffold Characterization

#### 2.6.1. Fourier Transform Infrared Spectroscopy with Attenuated Total Reflection (ATR-FTIR)

The procedure addressed in Section 2.3.1 for raw materials was also used for the scaffolds.

#### 2.6.2. Swelling Test

The gravimetric analysis method was used for all supports, both cross-linked and non-cross-linked, in water. Cylindrical portions of approximately 2 g were excised by transverse cutting. Their dry weight (*W_i_*) was recorded, and they were immersed in distilled water, with weight measurements taken at specified time intervals. The degree of swelling (*W*) was determined according to Equation (4), where *W_t_* is the mass of the scaffold at time *t*, and *W_i_* is the mass corresponding to the initial state.

(4) $$W=\frac{W_{t}-W_{i}}{W_{i}}=100\frac{W_{t}}{W_{i}}-100$$

#### 2.6.3. Sericin Release Study

The scaffolds were fully immersed in the sericin dissolution for three minutes in order to guarantee the maximum load of the protein. Then, the three-dimensional structures were immersed in 10 mL of distilled water, which was completely extracted at scheduled time intervals (0.2, 0.4, 0.6, 0.8, 1, 3, 5, 7, and 10 s), and then replenished to the same volume with fresh liquid. The release profile was determined by Ultraviolet-Visible spectrophotometry, using the conditions previously described in Section 2.3.7 at *λ* = 280 nm.

### 2.7. Statistical Analysis

For the statistical processing of the data and graphics, the software Excel from the Microsoft Office LTSC 2021 package (Microsoft Corp, King County, WA, USA), Statgraphics Centurion XVIII (StatPoint Technologies Inc., Warrenton, VA, USA), and OriginLab Pro 2024 (OriginLab Corp, Northampton, MA, USA) were used. Herein, the data are reported as mean ± standard deviation (SD), unless otherwise stated. Data were analyzed using Students’ *t*-test and two-way analysis of variance (ANOVA). In all analyses, a significant difference was inferred at *α* < 0.05.

## 3. Results

### 3.1. Materials Characterization

#### 3.1.1. Fourier Transform Infrared Spectroscopy with Attenuated Total Reflection (ATR-FTIR)

**Figure 1:**
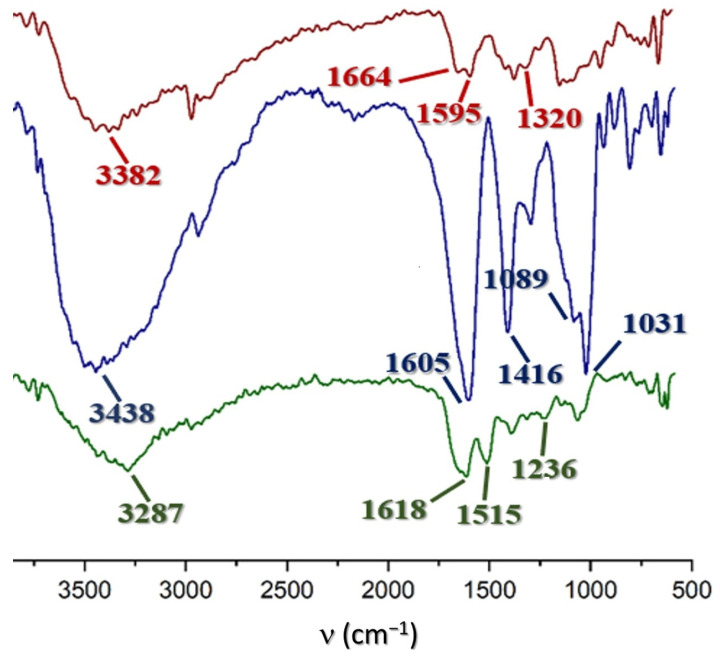
FTIR spectra for chitosan (red), alginate (blue), and sericin (green).

The FTIR spectra for chitosan, alginate, and sericin are presented in Figure 1. In all three cases, the coincidence of the absorption bands with the respective references was confirmed [13,14,15], which corroborates their chemical identity.

For alginate, a broad band was observed around 3438 cm^−1^, assigned to hydroxyl stretching vibrations ν(OHˉ). The appearance of two other intense signals around 1600 and 1400 cm^−1^ are related to asymmetric and symmetric stretching vibrations of carboxylate groups (COOˉ, stretching vibrations), respectively. The alginate FTIR spectra also showed the typical bands of alginic acid, which indicate the presence of mannuronic and guluronic acid at 887 and 945 cm^−1^, (bending vibration δ(CH)) which was obvious from the width of the hydroxylic vibration due to the dimerization of the acid group, respectively.

Chitosan presented bands located at 1664 cm^−1^ (stretching vibration, ν(C=O)) and 1595 cm^−1^ (stretching vibration, ν(C-N)) which correspond to amide I and amide II, respectively. These signals confirm the presence of amide groups, which were not eliminated in the deacetylation process of this material. The broad and intense band at 3382 cm^−1^ is assigned to the NH stretching vibration of the amino groups present in the rings of this polysaccharide. The presence of glycosidic bonds is corroborated by the band at 1076 cm^−1^ that corresponds to the COC stretching vibration. Finally, the bands at 2891 cm^−1^, 1420 cm^−1^, and 1325 cm^−1^ correspond to the stretching vibration ν^as^(C_sp_^3^-H) and bending vibration of the methylene, hydroxyl, and methyne groups, respectively.

For the sericin sample, the presence of vibrations of the characteristic bands of the amide groups in the proteins is evident: amide A and B (approximately at 3500 and 3000 cm^−1^, respectively), related to the stretching of the OH bonds (amide A) and the Fermi resonance among the first overtone amide II and N-H stretching vibrations (amide B). The amide I band (1600–1700 cm^−1^) represents the stretching of the C=O bonds, which are present in the main chain of the polypeptides and therefore more sensitive to the secondary structure and molecular orientation of the protein; amide II (1504–1582 cm^−1^) is associated with stretching of CN bonds and deformation of NH bonds. Finally, amide III (1200–1300 cm^−1^) is a very complex band and is associated with coordinate displacements related to superpositions of several bands in the fingerprint region of the spectra.

#### 3.1.2. Humidity Percentage

The average percentage of humidity obtained for chitosan was 14.7 ± 0.4%, consistent with another research [16,17]. However, this value is higher than those recorded by other authors [18,19]. Dissimilar values have been reported in the literature, generally ranging from 0.4 to 20%. The discrepancies in values across such a wide range can be attributed to several factors. Regarding the raw material, the source of chitosan extraction plays a crucial role. Even within the same species, such as crustaceans, chitosan can exhibit varying moisture levels. These variations are influenced by factors such as pH, environmental conditions, and temperature, all of which impact water retention. Additionally, the processing method significantly affects the final product. Differences in technological capabilities across industries can lead to variations in chitosan properties, particularly since it is derived from food processing waste. These factors collectively contribute to the observed discrepancies in values.

Average percentage of humidity obtained for alginate was 21 ± 1%. This commercial polymer was extracted from the species *Laminaria hyperborea*. There are reports of lower values within the range of 6.4–13.1% for the species *Padina boergesenii*, *Turbinaria triquetra*, *Hormophysa cuneiformis*, *Dictyota ciliolate*, and *Sargassum aquifolium* from the Red Sea, and 10.67% for *Sargassum latifolium* [13,20]. Thus, there is also variability in the moisture content of each species, with the source being one of the main factors influencing the physicochemical characteristics of the alginates obtained [18,19,21,22].

#### 3.1.3. Ash Content

The average ash percentage obtained for chitosan was 5.1 ± 0.6%, which implies a significant content of inorganic impurities. However, this is consistent with reports from several authors, which range from 0.4% to 29.2% [21,22,23,24]. This parameter is related to minerals present in the bodies of insects and crustaceans from which chitosan is extracted. Various studies have reported elements such as potassium, magnesium, sodium, iron, and calcium, which vary depending on factors such as size, order, sex, season, and subsequent conditions of slaughter [23]. This suggests a moderate effectiveness in demineralization given the low ash content. In any case, these traces are not recommended for this type of dermal application, so the low concentration is satisfactory to a certain extent, increasing the possibility of the sponge as a support for sericin solutions, which would be solubilizing said mineral traces and altering the protein concentration in the sponge [25].

In the case of alginate, the average ash content was 5.0 ± 0.4%. This value reflects the cations and anions produced by the organisms from which this product is derived. As a result of growing in a marine environment, the largest proportion corresponds to chloride and potassium [26].

#### 3.1.4. Alginate *M/G* Ratio

The *M/G* ratio obtained was 1.3 ± 0.2, similar to the results reported by Fertah and collaborators, suggesting that this system is more likely to form soft and elastic gels rather than brittle ones [27]. Gel strength primarily depends on the content and length of guluronic acid (*G*) in the alginate. Alginates high in guluronic acid are generally known to form strong but brittle gels, whereas those rich in mannuronic acid or mixed sequences yield weaker but more flexible gels. This is due to the alginate distribution known as egg box, where guluronic acid would form the vertical “walls” and mannuronic acid, the horizontal structures in the four arrangements of the polymer MM, MG, GG, and GM. The vertical position of guluronic acid, when it gels by exchanging Na^+^ for Ca^2+^, gives greater hardness to the gelled structure, while the horizontal conformation gives greater flexibility by rotating the structure on itself to achieve gelation [28]. Additionally, the *M/G* ratio is closely related to the antioxidant activity of sodium alginate; as the *M/G* ratio increases, so does chain mobility, enhancing antioxidant activity (more efficient radical elimination) [29].

#### 3.1.5. Deacetylation Degree of Chitosan

**Figure 2:**
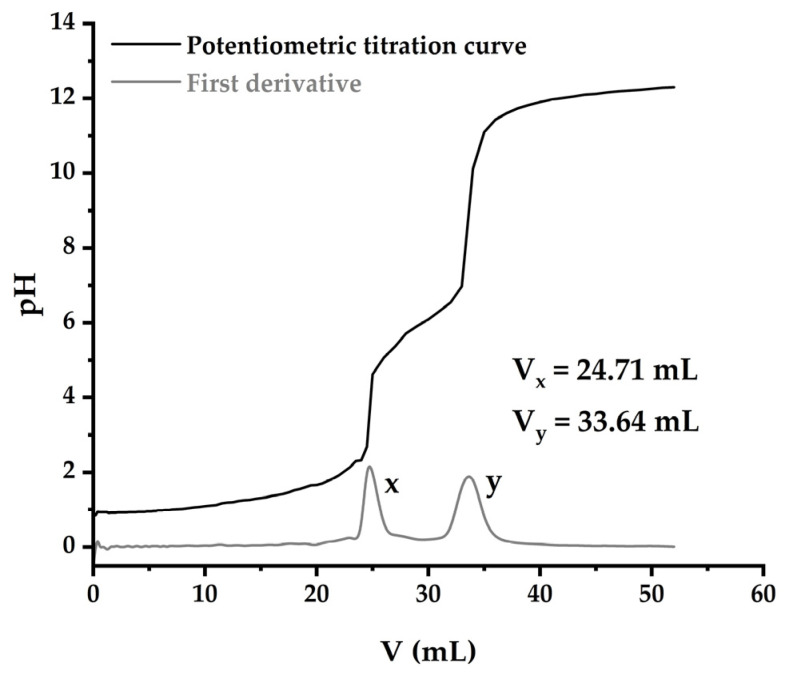
Titration curve and the first derivative of chitosan dissolution.

The exact titrant concentration was determined as 0.2426 ± 0.0006 mol/L. As shown in Figure 2, there are two equivalence points, and their values were calculated for each replicate. These volumes were associated with the first derivative of the equation obtained. The first equivalence point corresponds to the neutralization reaction of the known excess of HCl with sodium hydroxide; the second to the neutralization of the primary amino groups of chitosan, which are protonated at acidic pH and are deprotonated after the addition of a sufficient amount of base. The average degree of deacetylation of chitosan was 82 ± 2%, consistent with results obtained through the infrared spectroscopy method following the methodology proposed by Brugnerotto et al. [30].

The degree of deacetylation (DD) allows chitosan to be differentiated from chitin and determines its physical, chemical, and biological properties. This parameter is indicative of the balance between its repeating units (acetylated and deacetylated). Commercially, chitosan typically has a degree of deacetylation between 70 and 90%, while some biomedical applications require a DD over 95% [31]. The obtained value falls within the range 70–90%, similar to other commercial products.

The absorbances of certain reference bands were recorded, and using Equation (3), the DA and DD values corresponding to each replicate were calculated. The average degree of acetylation obtained by FTIR was 18 ± 7%, a value that agrees with the DD obtained by the potentiometric titration method, referred to in this section.

#### 3.1.6. Hydrodynamic Radius and Molecular Weight

Table 1 shows the value of the hydrodynamic radius and the molecular weight for alginate, chitosan, and sericin, obtained by the DLS and SLS techniques, respectively.

Alginate. The species of algae, as well as their age, geographical distribution, harvesting season, and extraction method strongly influence the molecular properties (molecular weight and composition) of the derived polymer [32]. Generally, the molecular weight of commercially available sodium alginates ranges between 32 and 400 kDa [33]. Therefore, the one corresponding to this study, with 85 ± 7 kDa, can be considered to have a low molecular weight, ranging within the usual values.

Chitosan. The molecular weight of chitosan can exceed 1000 kDa [34], according to which it is classified into three different types: low (<50 kDa), medium (50–250 kDa), and high (>250 kDa) molecular weight chitosan [35]. Therefore, the one in this study can be classified as low molecular weight chitosan (5.1 ± 0.2 kDa). On the other hand, while molecular weight significantly influences mechanical properties, its impact is less critical in our case due to the intended application of the scaffold. Our product is designed as a reusable sponge with high absorption capacity, which can be easily washed and reused for subsequent treatments, making mechanical strength a secondary concern.

For any polymer, solubility and viscosity depend on its molecular weight, but for chitosan, its effect extends to biological properties, so their determination becomes essential. For example, greater antibacterial activity has been reported in chitosan with low molecular weight compared to those with high molecular weight [36], as in the presented case.

Sericin. It has an average molar weight ranging from 0.3 to 400 kDa [37,38]. It is classified as high molecular weight if it exceeds 20 kDa, and low molecular weight if it is below this threshold [15]. In this study, a molecular weight of 57.4 ± 0.3 kDa was obtained, indicating that this sericin can be classified as high molecular weight.

Studies have reported that sericin with a molecular weight of 30–150 kDa cannot penetrate the cell membrane of the human epidermis, making absorption difficult by the skin. In contrast, sericin with a molecular weight between 12 and 17 kDa is suitable for cosmetic products for hair and nail care, while those in the range of 5–7 kDa are appropriate for body care. This smaller size allows for penetration of the cell membrane and complete absorption. Additionally, at this molecular weight, sericin exhibits properties that promote cell proliferation and inhibit cell death [15].

#### 3.1.7. Sericin Dissolution Characterization (Total Protein Concentration and pH)

**Figure 3:**
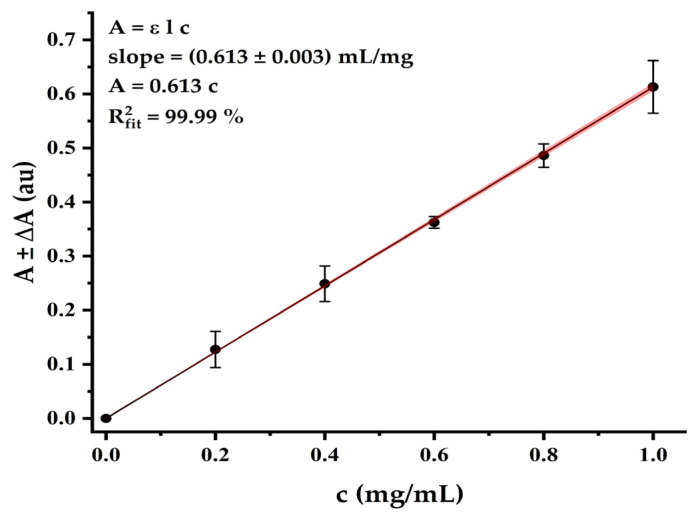
BSA calibration curve measured at 280 nm.

By measuring the absorbance values of each point of the BSA calibration curve at 280 nm, and performing an adjustment by linear regression, a representative graph was obtained, as shown in Figure 3. As can be seen, the straight model fits the experimental data, explaining 99.99% of the errors.

The absorbance of the sample dilutions was measured in triplicate, yielding a total protein concentration of 6.1 ± 0.3 mg/mL based on the straight-line equation. Since the composition of the silk cocoon consists primarily of fibroin and sericin, and there is a significant difference in their solubilities, it is anticipated that the soluble fraction obtained from this method will be composed almost entirely of sericin. Currently, in natural cosmetics, there are a variety of uses for sericulture products as ingredients, with their percentages in formulations varying significantly. Table 2 summarizes the permissible limits for some cosmetic products.

According to the information provided in Table 2, the silk sericin obtained is within the permissible limits for use in the specified cosmetic systems. If a reduction in concentration is necessary, dilutions can be prepared.

Depending on the desired effect as part of acne therapy, a satisfactory concentration for inclusion in a formulation can be established using the sericin obtained from silk degumming in this study. The anti-inflammatory, moisturizing, and photoprotective effects, along with its minimal toxicity, highlight the potential of the sericin solution as an active ingredient in dermocosmetic formulations for acne.

The pH determination revealed an average value of 6.31 ± 0.01, which falls within the recommended range for dermocosmetics intended for acneic skin (4.5–8) [39].

### 3.2. Scaffold Characterization

#### 3.2.1. Fourier Transform Infrared Spectroscopy with Attenuated Total Reflection (ATR-FTIR)

**Figure 4:**
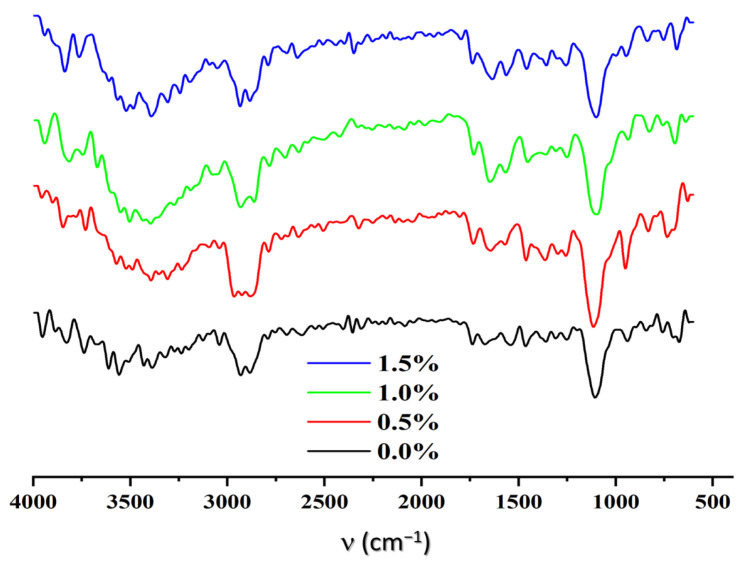
FTIR spectra of the ALG/CHI scaffolds.

The functional groups present in the structures of the chitosan and alginate sponges were identified by infrared spectroscopy, both for the matrices with varying degrees of cross-linking and those without it (Figure 4). These findings were compared with reference values from the literature [40].

As shown in Table 3, for all four sponges studied, the absorption bands of their spectra aligned with those of the raw material patterns used in their preparation. This corroborates the chemical identity of each scaffold.

In the case of chitosan, the spectrum includes bands at 1650 cm^−1^ [ν(C=O) (amide I)] and 1590 cm^−1^ [δ(NH_2_) (Amide II)]. In common with alginate, it presents the bands at 2920 cm^−1^ [ν(C_sp_^3^-H)] and 3430 cm^−1^ [ν(OH)]. Figure 4 also shows an increase in transmittance for the signal corresponding to the C=O stretching vibration in the cross-linked matrices.

#### 3.2.2. Swelling Test

**Figure 5:**
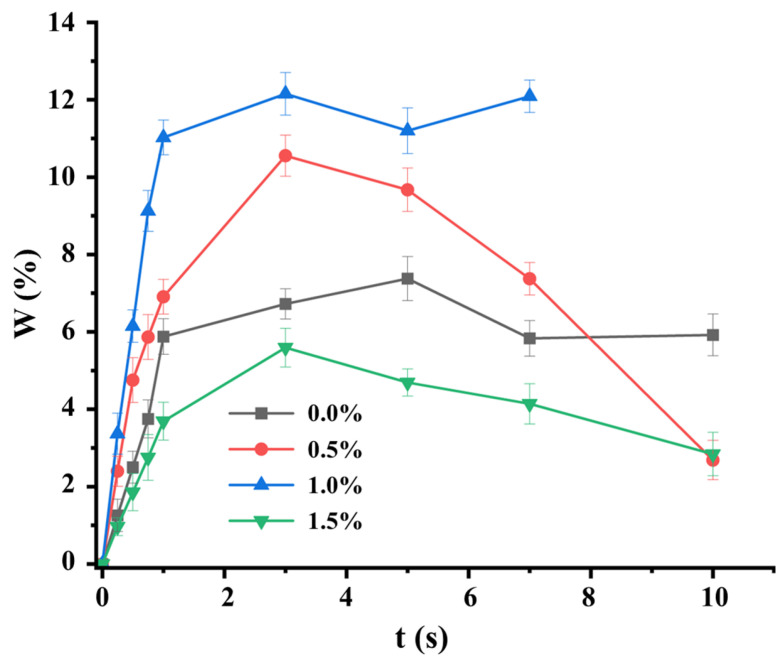
Swelling profiles of polymer matrices with different degrees of cross-linking.

An accelerated growth of material swelling is observed in all curves, reflecting the polymeric networks’ water absorption over time; followed by a plateau that indicates the maximum absorption capacity of the scaffold and relates microscopically to the chemical structure of the material (Figure 5) [41].

Notably, the material absorbs water very quickly; after just three seconds of immersion, it has already reached its maximum swelling capacity. The matrix retaining the greatest mass of water was the cross-linked sample with a 1% cross-linking grade, while the one that swelled the least was the one corresponding to 1.5%. This outcome aligns with literature findings, as increasing the degree of cross-linking of the chains reduces the free volume within the network structure, minimizing the size of the pores and the amount of fluid absorbed.

The latter, in a state of equilibrium, is a balance between the osmotic forces caused by the water when entering the macromolecular network and the cohesive forces exerted by the macromolecular chains that resist this expansion [42]. Furthermore, a network with a higher cross-linking density has less space available to be occupied by water and limited chain mobility, the elastic force that opposes swelling is increased [43].

However, the remaining samples did not follow the anticipated order for maximum swelling. This unexpected behaviour could be attributed to material characteristics, such as the number of layers, meaning that the layers involved according to the preparation process have an influence on the absorption capacity of the samples.

Given that the matrix is a hydrophilic chitosan and alginate polymer, the analysis began by evaluating the nature of the possible diffusion of the drug using the following Korsmeyer and Peppas equation [44]:

(5) $$\frac{M_{t}}{M_{\infty }}=k\cdot t^{n}$$

where *M_t_* and *M_∞_* are the amounts of drug released at time *t* and at equilibrium, respectively, *k* is the constant that depends on the shape and geometry of the drug carrier matrix, *n* is the diffusion exponent, and *t* is the release time.

**Figure 6:**
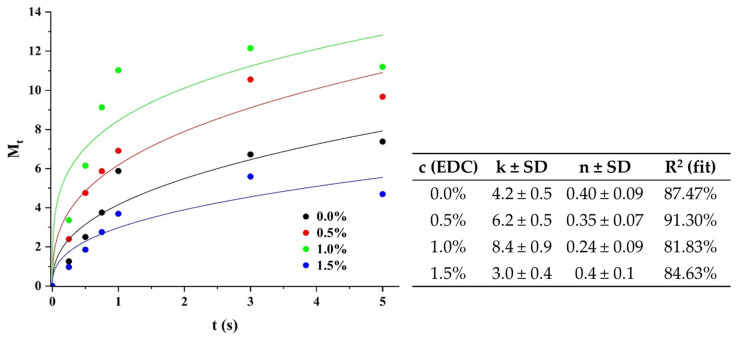
Fitting the swelling profiles and values of the main magnitudes obtained by the data-fit to the Korsmeyer–Peppas model.

Figure 6 shows the average parameters of the data fit to the Korsmeyer–Peppas equation, with their typical standard deviation (SD). The values of the diffusion exponent (*n*), in all cases, are less than 0.5, which indicates that this component is negligible in the system and the release is due almost entirely to the relaxation of the polymer chains.

**Figure 7:**
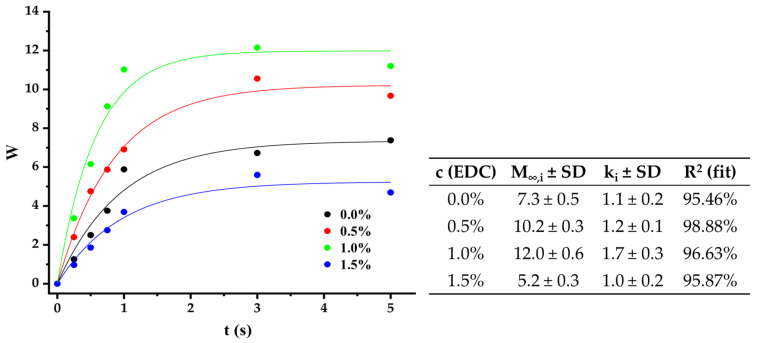
Graphics and values of the main magnitudes obtained by the data-fit to the Berens–Hopfenberg model.

To confirm the influence of chain relaxation on the swelling mechanism, the fit to the Berens–Hopfenberg equation was evaluated [45], which is commonly used to describe release from polymers that could degrade or erode during drug loss, regardless of their shape or dimensions (as the present case, Figure 7). This model is presented below.

(6) $$M_{t}=M_{\infty ,f}\left[1-\frac{6}{\pi^{2}}\sum_{n=1}^{x}\frac{1}{n^{2}}exp\left(-n^{2}k_{f}t\right)\right]+\sum_{i}^{k}M_{\infty ,i}[1-exp\left(-k_{i}t\right)]$$

where *M_∞,f_* is the amount absorbed at equilibrium, assuming that there are no relaxation processes in the material, *k_f_* is the constant of the diffusion process and depends on the diffusion coefficient and the “apparent” diameter of the particle, *M_∞,i_* is the amount absorbed at equilibrium, due only to the relaxation processes *i* in the material, *k_i_* is the rate constant of the relaxation process, and *t* is the measurement time.

**Figure 8:**
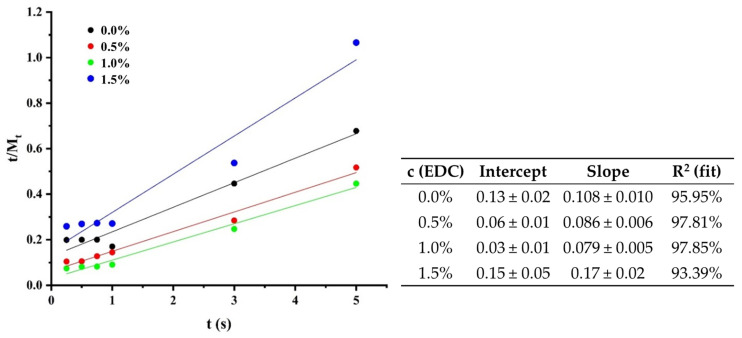
Graphics and values of the linear fitting of data in Equation (6).

The first factor of the equation expresses the contribution of diffusion in the swelling process and the second includes the relaxation processes typical of polymeric materials. For the fabricated scaffolds, the best fit was obtained by keeping only the second term, indicating that at least one type of relaxation process predominates in the swelling of the scaffolds. However, further trials are needed to confirm this conclusion and determine the nature of this process. In order to corroborate what has been stated so far about the swelling profiles, the fit to the pseudo-second-order model was evaluated (Figure 8) [46]. The model equation is presented below.

(7) $$\frac{t}{W_{t}}=\frac{t}{W_{\infty }}+\frac{1}{k_{W}W_{\infty }^{2}}$$

where *W_∞_* is the equilibrium swelling of the material, *k_W_* is the pseudo-second-order constant of the absorption process and *t* is the measurement time. As shown in Figure 8 and the values presented in table inside, the experimental data have a good fit to this equation, as represented by the values of the correlation coefficient. This last model confirms what was previously stated regarding the maximum swelling of the material, whose values coincide with the maximums of the swelling profile.

#### 3.2.3. Sericin Release Study

**Figure 9:**
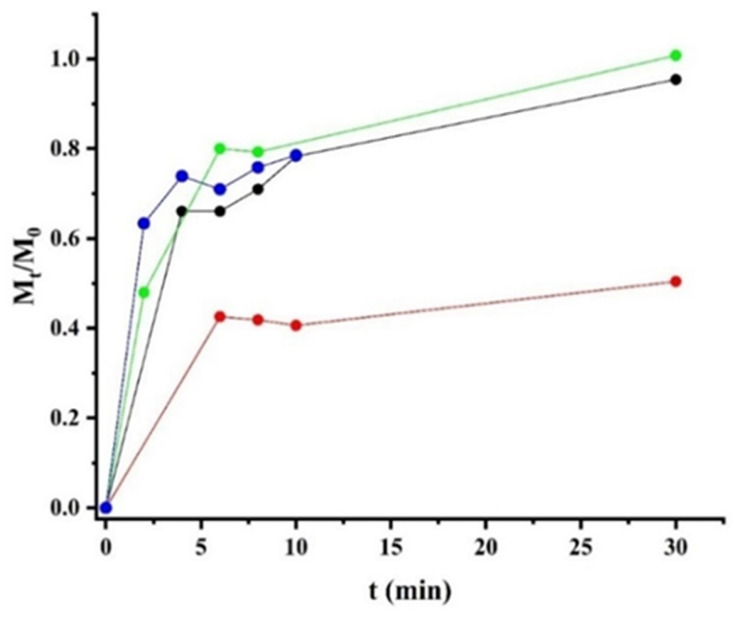
Release profiles of polymer matrices with different degrees of cross-linking: 0% (black), 0.5% (red), 1% (blue), and 1.5% (green).

Figure 9 shows the release profiles of the chitosan/alginate polymer matrices with varying degrees of cross-linking. Release studies are closely related to the behaviour of the swelling profile of polymeric matrices [47]. However, when making a comparison between the behaviour of the swelling degree and the sericin release in this study, there are marked differences in the time required to reach equilibrium and the distribution of data points in both graphs.

Typically, an increase in cross-linking density strengthens the polymer chains, resulting in a decreased swelling capacity [48]. Consequently, matrices with higher cross-linking absorb less liquid, which reduces the amount of liquid they can release. As shown in Figure 9, the release profiles did not follow a trend in terms of the maximum concentration of sericin released, mirroring the irregular pattern observed in the swelling profiles.

In all cases, the diffusion exponent (*n*) values were below 0.5, indicating that diffusion has a negligible role in the system, and the release is primarily governed by the relaxation of the polymer chains.

Additionally, as shown in Figure 9, the *M_i_* values do not follow the expected decreasing trend with an increase in the swelling degree from 0 to 1.5%.

### 3.3. Multivariate Statistical Analysis

In order to verify whether the behaviour of the physical–chemical parameters studied for each type of scaffold belonged to the same data structure, typical of similar microscopic processes, or if experimental errors might be present, Andrews curves were used construct a graph of these results.

Andrews curves are a valuable graphical statistical tool, which consists of the representation of multivariate data in a two- or three-dimensional space [49]. This method preserves data properties such as mean, variance, and distance [49,50,51], through the following function:

(8) $$f\left(t\right)=\frac{x_{1}}{\sqrt{2}}+x_{2}sen\left(t\right)+x_{3}cos\left(t\right)+x_{4}sen\left(2t\right)+x_{5}cos\left(2t\right)+\ldots$$

where *x_i_* represents the values of each variable for each sample studied, *t* is an arbitrary parameter (−π≤t≤ π), and *f*(*t*) is the corresponding function. Then, for each sample studied, a *f*(*t*) curve is generated based on each of the measured variables. With these functions, it can be noticed correlated observations (evident when multiple curves intersect frequently), outliers (distinguished by curves separated from areas of high curve density), and subgroups of data (which appear as clusters of curves, separated from others) [49].

Five variables studied and described in the previous sections from the Korsmeyer–Peppas equation were included, which characterize each type of cross-linked matrix: *Ge*, cross-linking degree; *k_W_*, swelling kinetic constant; *k_LIB_*, kinetic release constant; *n_W_*, diffusional swelling exponent; *n_LIB_*, diffusional liberation exponent.

For the use of the Andrews curves method, the order in which the values of the variables are assigned to the values of x_i_ is very important, assigning x_1_ to the variable contributing most significantly to data variance, and so forth [51].

Principal Component Analysis (PCA) was initially applied to determine the data structure (Table 4) of the samples, assuming four principal components can be studied only by taking the first principal component (PC1), which accounts for over 90% of the variance of the studied data.

**Figure 10:**
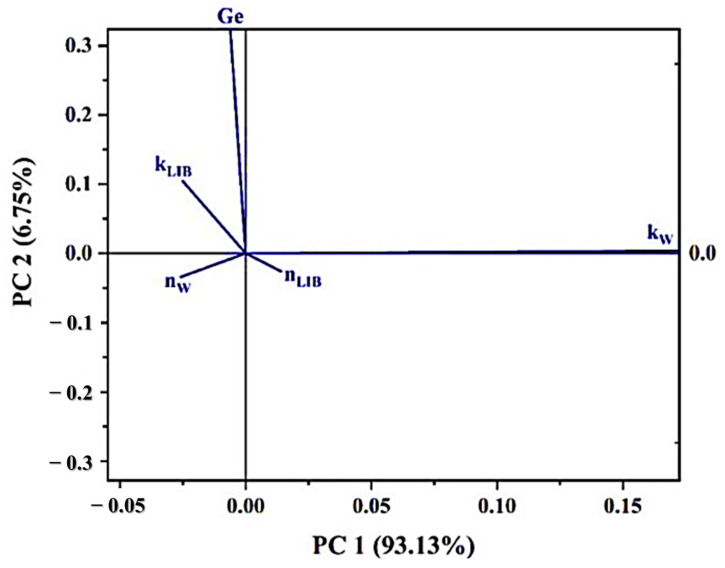
PCA biplot plot for the obtained matrices.

To know the degree of importance of each variable in the variance of the data set, the PCA biplot graph was analyzed (Figure 10), where the length of the projection on the PC1 axis of the vectors for each variable indicates the degree of importance of these in the total variance.

It was observed that *k_W_* > *k_LIB_* > *n_W_* > *n_LIB_* > *Ge* which allows the variables to be selected in that order to construct the Andrews curves corresponding to each sample, assigning *x_1_* to the variable *k_W_* (the one that most influences the total variance), and continues like this.

**Figure 11:**
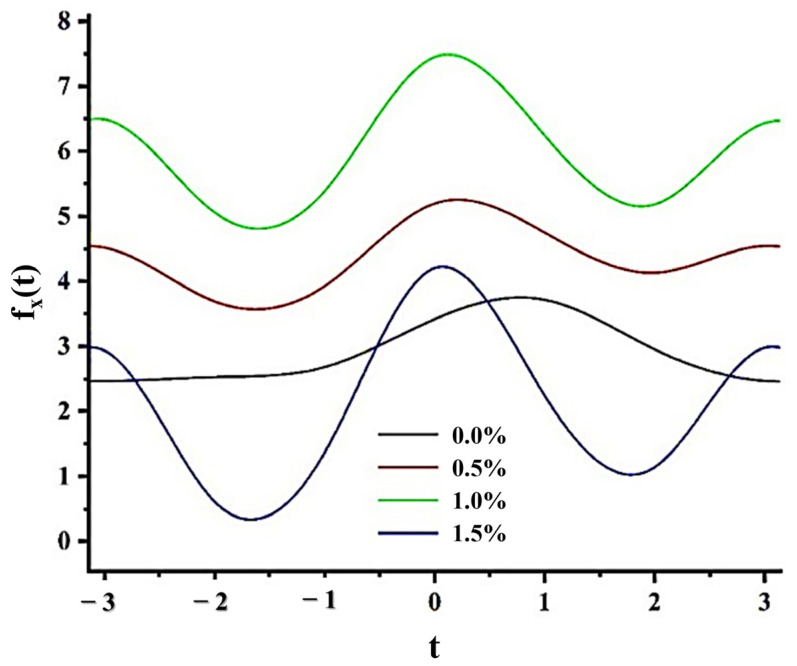
Andrews curves to study de influence of the crosslinking concentration.

Finally, the curves of each type of scaffold were constructed assuming a series of arbitrary parameters *t*, with −π ≤ *t* ≤ π; computing its image *f*(*t*) and plotting the points [*f*(*t*); *t*] (Figure 11).

In Figure 11, the four curves corresponding to each matrix type are shown. Notably, the appearance of a subgroup of curves corresponding to the three intertwined matrices stands out, which exhibit the same behaviour, where the maximums, minimums, and their frequencies approximately coincide.

From this result, it can be inferred that although the values of some variables for matrices with different degrees of crossing deviate from the expected trend, these data statistically align with the structure of the other scaffolds. Therefore, it can be stated that in these cases there are no outliers, and their deviations from the expected trend are not due to experimental errors. This result complements what was stated in previous sections on factors affecting the studied variables.

Finally, the curve corresponding to the non-crosslinked matrix presents a distinct behaviour from the rest of the matrices, which is to be expected given the influence of the crosslinking process itself on the release and swelling profiles.

## 4. Discussion

The band corresponding to amide I provides useful information about the protein’s secondary structure. Typically, the region from 1600 to 1640 cm^−1^ corresponds to intermolecular β-sheet bands, and the region from 1640 to 1660 cm^−1^ indicates random coils and α-helices. The remaining region from 1660 to 1690 cm^−1^ corresponds mainly to turns, indicating the existence of β-sheet or aggregate structure [52]. Since amide I is located at 1618 cm^−1^, it can be concluded that the-β sheet predominates in the secondary structure of the sericin under study [15].

This wide range is logical because the biopolymer humidity levels depend on factors such as the method, the extraction conditions, and the natural source used, which is generally of marine origin, but chitin has also been extracted from species of insects [53]. Furthermore, the great water absorption capacity of chitosan and the high percentage of relative humidity in the city of Havana (%RH > 75%) promote a high value of this parameter [54].

Water absorption kinetics in alginate biopolymers have been experimentally demonstrated to be dependent on temperature and relative humidity [55]. Then, since the country is situated in zone IV, with a hot and humid climate, a high value of residual humidity is to be expected.

The viscosity of alginates increases with longer molecules, higher concentrations, and a greater number of monomer units present in the alginate chains (i.e., the average molecular weight), since the longer the alginate chain, the greater the viscosity at similar concentrations [28,56]. Thus, this biopolymer likely has a medium chain length and medium/low viscosity, similar to that reported in other cases for the corresponding molecular weight [57]. Regarding its biological activity, its antioxidant properties are independent of its molecular weight, as various studies confirm [29]. Therefore, the values obtained from this property will not affect the polymer’s antioxidant capacity.

The greater the molecular weight, the greater the chain cross-linking and, as a consequence, water solubility decreases. In addition, the hydrophobic portion of the molecule increases, so the proportion of van der Waals interactions increases with respect to hydrogen bond interactions [58]. As it has a lower molecular weight, it presents less electrostatics interactions (van der Waals forces, hydrogen bridge, etc) in solution, resulting in more flexible chains that cause a reduction in viscosity [36].

When sericin has a higher molecular weight it is mainly used in biomedical materials, functional biomembranes, hydrogels, and in the production of functional fibres, and that with a lower molecular weight is frequently used in medicines and cosmetics [59]. Therefore, this product’s inclusion in a biomaterial is promising according to the behaviour of this property.

Although the sericin obtained does not have a low molecular weight, this problem can be solved by proposing a change in the extraction method for future research. For example, enzymatic hydrolysis could be carried out, a method that has been shown to be feasible to obtain sericin for cosmetic purposes [60].

Suppression of inflammation by sericin has been reported at concentrations of 0.004 to 1000 mg/mL. It is capable of increasing the amounts of inflammatory mediators and proinflammatory cytokines such as tumour necrosis factor α (TNF-α) and interleukin-1β (IL-1β), which participate in the modulation of skin growth, repair, and healing during inflammation. However, maximum levels of cytokines are insufficient to provoke an inflammatory response or inhibit cell proliferation [61,62].

In reported toxicological studies, sericin effects on skin depigmentation have been evaluated. For example, in a study by Aramwit et al., sericin was formulated as an 8% cream, observing a moisturizing and healing effect. Furthermore, at the end of the study, the level of skin pigmentation was significantly reduced; however, on a positive note, no toxic effects were demonstrated at this concentration [63,64]. Another study suggests that a topical sericin application (5 mg) has a photoprotective effect against acute UVB-induced damage and tumour promotion by reducing oxidative stress, cyclooxygenase 2 (COX-2), and cell proliferation in mouse skin.

Maintaining an optimal pH is crucial for healthy and protected skin. Products with a slightly acidic or neutral pH, like this one, are most appropriate for people prone to seborrheic-type diseases (acne vulgaris, rosacea), atopic skin, irritant dermatitis, contact dermatitis, and ichthyosis. A high skin pH can influence the enzymatic activity of lipid metabolism in the stratum corneum, compromising the acid mantle and, therefore, providing a favourable environment for the growth of particularly harmful bacteria such as *Staphylococcus aureus* [65].

The formation of amide-type bonds between the COOH groups of the alginate and free NH_2_ of the chitosan is facilitated by the EDC/NHS cross-linking reagent. In this way, the increase in signal intensity should be a measure of the efficiency of the cross-linking process.

Chitosan and alginate sponges are widely used in tissue engineering, where materials with adequate mechanical properties are essential for implantation [40]. Therefore, it is generally required that they have a certain thickness, degree of cross-linking, and the number of layers, which simulate the concentration gradient [66]. However, in this case, with the aim of obtaining a less rigid material for use in cosmetics, it was decided to prepare a monolayer scaffold, which would also result in a suitable matrix for drug release. Despite not conducting formal mechanical property tests, it was possible to verify by handling the sponges that, in all cases, were fragile and erodible, which confirms that the expected rigidity was not obtained by the cross-linking. This implies that, upon swelling, the mechanical resistance that the chains will offer will not be entirely controlled by the chemical structure of the material, which explains why there is no tendency to increase the degree of cross-linking, rather an oscillation around a maximum value of swelling.

This could be attributed, firstly, to the presence of a solute (sericin) in the case of release, which allows controlling the flow in and out of the matrix due to concentration. By contrast, when evaluating swelling, distilled water was used, which could cause fluctuations in solvent retention with different *Ge* [67].

It should also be considered that the error of the technique is greater in determining the swelling degree than in obtaining the release profile. The first is carried out by the difference in weight, much less accurate than determining the concentration by measuring the absorbance at 280 nm, when the small mass of the scaffold is used in each measurement and the lower reproducibility of the technique is taken into consideration.

This behaviour suggests that the degree of physical cross-linking achieved through the manufacturing process is insufficient to impart the porous platform with the ability to control the chain relaxation process, until a higher degree of cross-linking is achieved through the use of chemical agents such as EDC/NHS [68].

Furthermore, the incorporation of the drug adds one more variable to the process control, which can make biassed behaviour difficult with increasing cross-linker concentration. As had been analyzed in the section corresponding to the swelling study, the lack of regularity when incorporating the drug may be associated with the increase in fluid concentration. Meanwhile, the rest, including the non-cross-linked one, do not show significant differences in their behaviour.

This performance is in accordance with the results of swelling, since the amount of liquid absorbed, solely due to the relaxation process of the chains, is proportional to the total amount of liquid that the material is capable of absorbing.

## 5. Conclusions

A monolayer porous scaffold made of alginate and chitosan and doped with sericin for the dermocosmetic treatment of acne was designed and characterized, and preliminary evaluations were conducted. The sericin obtained had a mass of 57.4 kDa and was obtained with a concentration of 6.1 ± 0.3 mg/mL. The scaffold showed a great capacity for swelling and fluid retention and the statistical evaluation of these parameters was satisfactory. The release of sericin occurred through polymeric relaxation mechanisms certified by the adjustment to the second term of the Berens–Hopfenberg equation. Finally, it was possible to verify by multivariate analysis the order of importance of the variables involved in the study, corroborating again the significance of the parameters related to polymeric relaxation as the most determinant in the behaviour of the scaffold.

## Figures and Tables

**Table 1:** Hydrodynamic radius (*R_h_*) and molecular weight (*MW*) of the polymers used.

Polymer	*R_h_* (nm)	Molecular Weight (kDa)
Alginate	170 ± 20	85 ± 7
Chitosan	36.3 ± 0.2	5.1 ± 0.2
Sericin	32 ± 4	57.4 ± 0.3

**Table 2:** Sericin concentration in cosmetics.

Cosmetic	Limit Concentration (*w*/*w*)
Sericin powder	5–30%
Lotions and creams	0.001–30.00%
Nail cosmetics	0.02–20.00%
Bath and hair preparations	0.02–2.00%

**Table 3:** Assignment of the main FTIR bands of the scaffolds.

ν_ref_ (cm^−1^)	ν_exp_ (cm^−1^)				Assignment
	0.0%	0.5%	1.0%	1.5%	
3430	3430	3394	3394	3394	ν(OH); ν(NH)
2920	2931	2924	2932	2934	ν(C_sp_^3^-H)
1650	1672	1648	1648	1634	ν(C=O) [Amide I]
1590	1544	1566	1566	1566	δ (NH_2_) [Amide II]

**Table 4:** Data on the variables selected for each scaffold.

*Ge*	*k_W_*	*k_LIB_*	*n_W_*	*n_LIB_*
0	4.156	0.48	0.40	0.20
0.5	6.184	0.323	0.35	0.13
1	8.448	0.476	0.26	0.23
1.5	2.979	0.597	0.39	0.12

## Data Availability

Data are contained within the article.
